# British Gynæcological Society

**Published:** 1904-01-16

**Authors:** 


### Editor's Letter-Box.

**[Our Correspondents are reminded that prolixity is a great bar to publication, and that brevity of style and conciseness of statement greatly facilitate early insertion.]**

### BRITISH GYNÆCOLOGICAL SOCIETY.

In asking us to announce last week the date of their next examination for certificates in maternity nursing and in gynæcological nursing, to be held in the first week of March, the British Gynæcological Society requested us to insert the following:—

"Many nurses do not appear to realize the importance of the Midwives Act of 1902 to themselves. A large section of them desire to take up monthly nursing, and doctors require that they should possess some special certificate of efficiency in that branch of work. Hitherto, of course, the only independent certificate which they could obtain was that given by the London Obstetrical Society, which certified as to their competency as 'midwives.' The Midwives Act has created a sharp line of division between those who desire to be registered as midwives, that is to say who desire to work as independent practitioners under the Midwives Board, and for the most part amongst the very poor, and those who desire to do maternity nursing under the supervision of medical practitioners, and, for the most part, amongst the middle and upper classes. The former class require a certificate of efficiency as a midwife; for the latter class such a certificate is of no value; whilst an independent maternity nursing certificate would doubtless be valuable. In fact, the time has come when such nurses must make their decision whether they intend to be midwives and be so registered, or whether they desire to work as monthly nurses; because, in the former case they will act as competitors with medical men, in the latter case only as the doctors' assistants; and it can hardly be expected that medical practitioners will employ a woman who may be an active competitor with themselves, to act as a monthly nurse under their direction. In short, the nurse who becomes registered as a midwife obtains a definite legal status as a practitioner of midwifery; but, on the other hand, she is not likely to receive any assistance or work from medical practitioners as a monthly nurse."

